# From strategy development to routine implementation: the cost of Intermittent Preventive Treatment in Infants for malaria control

**DOI:** 10.1186/1472-6963-8-165

**Published:** 2008-07-31

**Authors:** Fatuma Manzi, Guy Hutton, Joanna Schellenberg, Marcel Tanner, Pedro Alonso, Hassan Mshinda, David Schellenberg

**Affiliations:** 1 Ifakara Health Research and Development Centre, Ifakara, Tanzania; 2 Swiss Tropical Institute, Basel, Switzerland; 3 London School of Hygiene and Tropical Medicine, London, UK; 4 Centre for International Health, Institut de Investigaciones Biomedicas August Pi I Sunyer (IDIBAPS), Barcelona, Spain

## Abstract

**Background:**

Achieving the Millennium Development Goals for health requires a massive scaling-up of interventions in Sub Saharan Africa. Intermittent Preventive Treatment in infants (IPTi) is a promising new tool for malaria control. Although efficacy information is available for many interventions, there is a dearth of data on the resources required for scaling up of health interventions.

**Method:**

We worked in partnership with the Ministry of Health and Social Welfare (MoHSW) to develop an IPTi strategy that could be implemented and managed by routine health services. We tracked health system and other costs of (1) developing the strategy and (2) maintaining routine implementation of the strategy in five districts in southern Tanzania. Financial costs were extracted and summarized from a costing template and semi-structured interviews were conducted with key informants to record time and resources spent on IPTi activities.

**Results:**

The estimated financial cost to start-up and run IPTi in the whole of Tanzania in 2005 was US$1,486,284. Start-up costs of US$36,363 were incurred at the national level, mainly on the development of Behaviour Change Communication (BCC) materials, stakeholders' meetings and other consultations. The annual running cost at national level for intervention management and monitoring and drug purchase was estimated at US$459,096. Start-up costs at the district level were US$7,885 per district, mainly expenditure on training. Annual running costs were US$170 per district, mainly for printing of BCC materials. There was no incremental financial expenditure needed to deliver the intervention in health facilities as supplies were delivered alongside routine vaccinations and available health workers performed the activities without working overtime. The economic cost was estimated at 23 US cents per IPTi dose delivered.

**Conclusion:**

The costs presented here show the order of magnitude of expenditures needed to initiate and to implement IPTi at national scale in settings with high Expanded Programme on Immunization (EPI) coverage. The IPTi intervention appears to be affordable even within the budget constraints of Ministries of Health of most sub-Saharan African countries.

## Background

A massive scaling-up of health interventions is required over the coming years[1] if sub-Saharan Africa is to reach the health-related Millennium Development Goals (MDG). Efficacy information is available for key interventions[2] but there is a dearth of information on the resources required for implementation of interventions known to be cost-effective. In recent years costing information has been an integral part of health intervention evaluation as it is vital for management, prioritization and scaling-up of health interventions[3].

Successful scaling-up of a health intervention is based on two prerequisites: first is the *development *of an effective delivery and management system at various levels and second is the longer-term *implementation *of the intervention based on a good knowledge of costs and sound financing strategies. In this study, we costed the resources required to set up integrated management and implementation systems for an intervention to use an existing platform of routine health service delivery. In recent years economists have published a number of costing studies of malaria control interventions, some concerning new interventions[4], some on the cost of existing interventions [5-8] and others on program administrative cost or policy change [9-11]. Few published studies[4,10] estimated both the cost of developing a delivery strategy and the incremental costs of incorporating an intervention into the routine health system.

Intermittent Preventive Malaria Treatment in infants (IPTi) is a promising tool to fight malaria [12-16]. Efforts are underway through the IPTi Consortium to generate evidence on the safety, efficacy, acceptability and cost-effectiveness of IPTi in a range of settings so as to inform a policy recommendation . If IPTi is recommended as a policy, the next challenge will be to transform this intervention into public health action in a reasonable timeframe. This requires a well developed delivery strategy and information on resources required.

In Southern Tanzania, local IPTi safety and efficacy data were available in 2001[15] and there was interest to explore the operational issues surrounding large-scale implementation as part of an integrated district-based approach to control malaria. Together with the Ministry of Health and other partners, a strategy for the delivery of IPTi was developed, implemented and evaluated between 2004 and 2008. These activities enabled a parallel costing study to produce realistic estimates of the costs of developing the delivery strategy, and of implementing and maintaining it. In contrast to many costing studies, few assumptions were needed as economic costs were simply tracked during the development and implementation phases. For example, we included administrative costs important for decision making and implementation such as stakeholder consultations, adapting the health information system and developing behavior change communication materials for the strategy. The study was designed to enable us to answer the question "What resources are needed to implement IPTi?", something asked by policy implementers at district level, such as Council Health Management Teams (CHMTs) in Tanzania. This study therefore provides insights into the budget implications of scaling-up IPTi and prepares the way for identifying future financing options.

## Methods

### Background

A Tanzanian district is administered at the 3rd level of the government hierarchy, and is also called a Local Government Authority. The district's main purpose is to empower the people to participate in the planning and implementation of local development programmes. In terms of health objectives, a district is a focal point for the planning, delivery and evaluation of health services. Districts mobilize, manage and account for government health resources and deliver health services in line with their own plans and budget allocations.

Health financing in Tanzania involves central government (Ministry of Health and Social Welfare – MoHSW), households, donors, NGO's and private firms. In 1999/2000, households provided the greatest proportion of health care financing (47%), followed by donors (23%), government (22%), NGOs (5%) and firms (3%)[17]. Government spending on health in Tanzania has increased in recent years standing at US$369 million in 2006 (US$ 9.92 per capita) which represents 11.6% of overall government spending (Table 1)[18]. Health sector spending is financed from block grants which are sourced from the Tanzanian government and the health 'basket' fund which is contributed to by donors.

**Table 1:** Health resources in Tanzania for year 2003 – 2006

**Item**	**Year**			
	
	**2003**	**2004**	**2005**	**2006^a^**
Health as percentage of overall government spending^b^	12.9%	10.0%	10.9%	11.6%
Level of spending on health (millions)	US$172	US$200	US$261	US$369
Population estimates	34,155,840	35,146,359	36,165,604	37,214,406
Per capita health spending	US$5.04	US$5.70	US$7.21	US$9.92

^a^Budget ^b^Excluding consolidated fund services (CFS).

Source: United Republic of Tanzania. Ministry of health. Health sector PER update FY06. Final Report. September 2006.

A Comprehensive Council Health Plan (CCHP) brings together annual plans and details the provision of accessible, quality health care services for local communities. These include cost effective interventions developed according to the Essential Health Package (EHP) and in line with the National Health Policy. The CCHP is supported by Local Health Block Grants and the Health Basket Grants based on criteria of population size (70% of the grant value), vehicle mileage (10%), poverty status (10%) and under five mortality rates (10%)[19]. These criteria are important in the sense that the individual is recognized as the main client of the health service, with children aged under five years and pregnant women recognized as the most vulnerable for many health issues. The mileage criterion takes into account the higher operational cost of delivering health services to a rural population and to scarcely populated areas, including the higher costs faced in drug distribution, immunization services and supervision. The under-five mortality criterion directs increased resources to places with higher burden of diseases. The CCHP is drafted by the planning team at the district level and then passed to the Regional Secretariat before approval is sought from the district council, the President's Office for Regional Administration and Local Government and the MoHSW. At the national level, checks are made for adherence to national guidelines, to make sure the plan meets both financial and technical performance requirements for funding.

### Study area

The Southern Tanzania IPTi effectiveness study is being conducted in five districts across two regions: Lindi Rural, Nachingwea and Ruangwa districts in Lindi region, and Tandahimba and Newala districts in Mtwara region. A detailed explanation is given elsewhere[20]. Briefly, the population of this poor, rural area is around 900,000 and subject to the highest infant mortality rate in the country at around 121 per 1,000 live births compared to national average of 68 per 1,000 live births[21,22]. Like the rest of Tanzania, the health system in the project area comprises a network of tiered public and private facilities where IPTi is delivered alongside Expanded Programme on Immunization (EPI) services. The EPI vaccine coverage is high, with 80–90% of all children aged 12–23 months receiving BCG, DPT and OPV before one year of age [20]. In 2006 the health facilities in the project area comprised seven hospitals, 13 health centres and 131 dispensaries. The project districts are further divided into 24 divisions and IPTi implementation was initiated in half of these divisions. National policy dictates that children and pregnant women should not pay for health services.

### The intervention and the delivery strategy

This study was a large scale community-randomised trial, which compared sets of process and outcome indicators between 12 IPTi-implementing divisions and 12 comparison divisions. The IPTi *intervention *is the delivery of a treatment dose of sulphadoxine-pyrimethamine (SP) to infants attending Reproductive and Child Health clinics for vaccination at three points in time: doses two and three of DTP/hep B/OPV, and the measles vaccine, corresponding to approximately 2, 3 and 9 months of age. An infant is given a quarter tablet of SP if they weigh under 5 Kg or a half tablet if they weigh 5 kg or over.

In order to achieve high and sustainable coverage of IPTi as part of routine preventive health services for infants, a number of elements are required that together constitute the IPTi *strategy*. The IPTi strategy was developed in close collaboration with the Ministry of Health and Social Welfare (MoHSW) to ensure that it would be feasible to roll out across the whole country should a policy decision be taken to implement IPTi nationally. The Expanded Programme on Immunization (EPI) which is well established in Tanzania was used as the vehicle for IPTi delivery. IPTi has been implemented by routine health workers. Routine health workers were trained to explain the IPTi intervention to mothers attending the EPI clinic with their infants, to raise community awareness on intervention more generally, and to deliver and document doses of IPTi using modified EPI monitoring tools alongside vaccinations in health facilities. The CHMT through the District Cold Chain Officer and Reproductive and Child Health Coordinator were equipped with adequate and specific tools to manage the requisition and supply of IPTi-related materials.

#### Costing: methods

One important distinction made in this study is between two types of health system cost: the cost of developing a delivery system for IPTi, which is mainly incurred at national level, and the cost of implementing IPTi, which is mainly incurred at the district level and below.

Costs were classified into different components, namely strategy development and sensitization; BCC material development; SP purchase and distribution; training; administration of the intervention in health facilities, and strategy management. A description of each is provided in Table 2.

**Table 2:** Components of implementation

1. Policy change and Sensitization
Policy change activities include planning, policy related consultations. Sensitization activities include meetings with stakeholders. Both policy change and sensitization involves working with a broad group of stakeholders at national and international organizations, a more focused IPTi core group of key stakeholders and district level health managers. These costs were incurred at the start of implementation.

2. Development of Behaviour Change Communication (BCC) materials

This was incurred before the start of implementation, but also includes minor recurrent costs related to occasional replacement of materials. Activities include development of materials (eg training leaflet, job aid, and posters), pilot testing, production and distribution.

3. SP purchase and distribution

This is mainly a recurrent cost. Purchase activities include importation and overhead costs of arranging importation. Distribution activities involve the distribution from port to Medical Stores Department (MSD), then to regional level, district level and finally to health facilities.

4. Training

This is mainly incurred at the start of implementation but also includes refresher and new staff training. The training involves training trainers at regional and district levels in strategy change, BCC and IPTi administration. In turn, these trainers train the front-line health facility staff.

5. Administration of the intervention in health facilities

This is a recurrent cost. It involves SP provision: preparation, administration to children, recording dosage and dates in immunization cards and books of the Health Management Information System (HMIS) by health workers at facilities. It also includes education of mothers about IPTi. This was calculated as the proportion of an RCH nurse's gross salary spent on IPTi per year at implementing facilities in the 5 study districts.

6. Strategy management

This is incurred partly at the start of implementation, and partly as an ongoing activity. The start-up costs are converted to annual costs assuming a 10 year intervention period. In the case of the Southern Tanzania project, it involved the recruitment of a public health professional to support the implementation activities. It also included consultations with regard to adaptation of HMIS and immunization cards, as well as printing costs.

Note that components 1 and 2 are sometimes referred to as administrative or higher level costs. They involve activities to get the intervention developed and implemented by the routine health system. The costs were spread over 10 years which is the expected lifetime of a national program

District level total costs and IPTi delivery unit costs were estimated from the health system perspective. Four different health system levels were considered – national, regional, district and health facility. However, regions have only a minor role in health system financing and that role is closer to the district than national role, and thus we summarized regional cost estimates to district level. District implementation costs thus reflect activities undertaken at regional, district and health facility levels. National level costs include all activities organized by the MoHSW and are mainly related to policy matters. Given that a scaled up strategy would be country-wide, the estimated costs of strategy change were allocated across the total number of districts in Tanzania. IPTi dose forecasts were based on current EPI coverage levels.

A costing template enabled detailed costs to be captured relating to each IPTi development and implementation activity. Expenditure on formal meetings and training was tracked by use of specific cost centres in the accounting software which were later reviewed to extract financial costs. Semi-structured interviews were conducted with nine key informants involved in early IPTi activities including formal meetings, informal meetings and independent desk work. These were IPTi investigators and support staff who were interviewed by one of us (FM) at their convenience according to a discussion guide. The reported time spent was used to derive estimates of financial and opportunity costs related to human resources for IPTi strategy development and implementation. Informal activities included consultations with key stakeholders which did not involve hiring meeting rooms. Desk work included the value of time spent by individuals in preparation, designing, brainstorming or organizing project activities. A summary sheet (table 2) was used to guide summarizing of data according to the activity components and health system level, with a distinction made between financial and non-financial 'opportunity' costs.

Opportunity costs were those that were diverted from other uses or were not fully employed and therefore using up slack (inefficiencies) in the system. Financial costs were those that involved direct budgetary impacts – in other words, financial payments. This cost classification was important with regard to using our estimates in other settings, as health systems financing and configuration differ widely across sub-Saharan Africa. Whereas in one setting the health system might have in-house expertise at the Ministry of Health, others might need external expertise – with a need for additional financial resources. The total cost, which is the estimated economic cost, is the sum of financial costs and non-financial (opportunity) costs. Cost classification was based on how related activities are usually done, and how they are valued, under a MoHSW-led program. There were no fixed assets purchased specifically for IPTi, but we accounted for hired fixed assets under transport and buildings. The costing estimates reflect a one year time frame.

#### Ethical clearance

This study received ethical approval from the institutional review boards of the Ifakara Health Research and Development Centre, the National Tanzania Medical Research Co-coordinating Committee, the Tanzania Commission for Science and Technology, and the London School of Hygiene and Tropical Medicine. During field work, information sheets about the study in Swahili were distributed, explaining why it was being carried out, by whom, and what it would involve. Verbal informed consent was obtained from all study participants and confidentiality was assured.

#### Sensitivity analysis

This costing study started at the beginning of the main IPTi project in 2004 and was completed in 2007. Excel spreadsheets were used to conduct sensitivity analyses on key variables that were susceptible to change over time or in different settings. These include EPI coverage, SP brand, inclusion of community sensitization (differentiating the intervention delivery by researchers and that by MoHSW), and changes in human resource cost due to recent increases in per diem rates and salaries for health staff in Tanzania.

## Results

The costs per district of developing the IPTi strategy and of implementation for the first year, are summarized in additional file 1. This total cost is broken down into the apportioned cost per district of national level activities and the cost per district of activities at district level or below. Costs were split into personnel, transport and materials and buildings.

The single largest expenditure was for training which cost US$7,392 per district, 51% of the total start-up and year one costs. There was no incremental financial expenditure needed to deliver the intervention in health facilities as available health workers performed the activities without working overtime. The other major intervention related cost was for drug purchase and distribution, some US$3,538 per district (24%). National development related costs were relatively modest when shared amongst the districts; Behaviour Change Communication (BCC) cost US$175 per district (1.2%). Simulated policy change and sensitization was US$56 per district (0.4%) and management and monitoring cost US$309 (2%) per district.

In terms of cost category, personnel costs amounted to US$6,693 (46% of the total cost), mainly for training (US$4,285) and sensitization (US$1,549). Of the total personnel cost, US$3,746 (56%) was incremental financial expenditure. The cost of transport was US$3,949 (27% of the total cost), again mainly for training (US$2,746) and sensitization (US$984). Materials and building costs accounted for US$3,944 (27% of the total costs).

Overall, approximately 28% of the costs per district were for national level activities and the rest were costs per district of activities at district level or below.

The unit costs are presented in Table 3. The total cost per IPTi dose delivered was estimated at 23 US cents, of which 18 US cents (78%) was financial expenditure and 5 US cents (22%) was opportunity cost.

**Table 3:** Estimated unit cost of IPTi per dose delivered. Figures are United States cents, year 2005 (Tsh1205 = US$1)

**Activity component**	**Health** **system** **level**	**Financial ** **costs**	**Opportunity ** **costs**	**Total costs**
Policy change	National	0.01	0.02	0.03
Sensitization	District	0.76	1.12	1.88
BCC	National	0.03	0.05	0.08
SP purchase and distribution	National	12.56	0.26	12.82
Training	District	3.06	2.30	5.36
Administration of intervention in health facilities	District	0.00	1.25	1.25
Strategy management	National	0.65	0.10	0.75
	District	0.62	0.00	0.62
***Sub-Total***	***National***	***13.25***	***0.43***	***13.68***
	***District***	***4.44***	***4.67***	***9.11***

**Overall total**		**17.68**	**5.11**	**22.79**

Table 4 summarizes the estimated financial start-up and annual running costs of IPTi, first at national level, second for a single decentralized district, and third to scale up to the123 districts in the whole country. At national level, the start-up cost was estimated at US$36,363 involving development of the strategy including BCC materials, stakeholders meetings and consultations. The annual running cost at national level for intervention management and monitoring and SP drug purchase was estimated at US$459,096. The start-up cost was US$7,885 per district, mainly for training of front-line health staff, with an annual running cost of US$170 per district largely for the printing of BCC materials. Overall US$1,006,278 would be required to develop the IPTi strategy and implement it in the whole country with an additional US$480,006 required to run the intervention across the country for a year.

**Table 4:** Summary of estimated financial costs for a national IPTi program in Tanzania: start-up and annual implementation.

***Level***	***Start-up costs *** ***(US$ in 2005)***	***Running cost *** ***(US$ in 2005)***	***Cost of IPTi strategy *** ***development & first *** ***year implementation*** ***(US$ in 2005)***
Cost at National level	36,363	459,096	69,092
Cost per district	*7,885*	*170*	*11,522*
Sub-total for 123 districts	969,915	20,910	1,417,192

Total for national implementation	**1,006,278**	**480,006**	**1,486,284**

Figures are United States dollars, year 2005 (Tsh.1205 = US$1)

### Sensitivity analysis

Table 5 shows the results of sensitivity analyses testing the impact of varying key inputs on the economic costs in the program. Normally under large scale MoHSW-run programs, community sensitization is not included, especially for introducing a new intervention into the EPI program. But since the intervention was implemented through a research programme, it was necessary to introduce researchers through meetings with district health management teams, district heads of departments and community representatives (councilors). These sensitization activities had some impact on the economic cost, with an increase of 8%.

**Table 5:** Results of sensitivity analysis on the economic cost per IPTi dose delivered. Figures are United States cents, year 2005 (Tsh1205 = US$1)

**Variables**	**Financial**	**Opportunity**	**Economic cost (Total)**
Base case results	17.68	5.11	22.79
Low EPI coverage (71% nationwide)	23.41	6.76	30.18
Exclude sensitization at community level	16.92	4.00	20.9
Use of local brand SP Drug	8.44	4.85	13.29
Increase in salaries and per diem	20.97	5.74	26.71
Using generic drug, increasing salaries, excluding sensitization and lower EPI coverage	22.40	5.28	27.68

The change in human resource remuneration following a salary increase of 50% for health ministry staff between 2004 and 2006 led to a 17% increase in economic costs per dose delivered. Reducing the intervention coverage from 94% (as reported in the 2006 EPI report[23]) to 71%(as reported by the Demographic and Health Survey 2005 [24]) led to a 32% increase in costs per dose delivered due to the spreading fixed national and district costs among fewer IPTi recipients. The use of locally manufactured SP had a major impact on unit costs, with unit cost per dose delivered reduced by 42% from 23 to 13 US cents, most of which was a saving in financial expenditure. Finally, the most likely scenario of using generic drug, increasing salaries, excluding sensitization and lower EPI coverage increased economic cost by 21%.

## Discussion

This paper has presented the total costs of developing and implementing a strategy for the delivery of IPTi. The information is based on the actual costs incurred in the development of an IPTi strategy in conjunction with national authorities and implementation by district health teams. This approach has the advantage of making very few assumptions as costs were tracked in the course of actual implementation. The results should nevertheless be interpreted with caution. The study activities were undertaken by researchers, and although an attempt was made to exclude irrelevant research costs, it is possible that the researchers influenced programme costs in comparison to government-led implementation. However, we are confident that the cost tracking system was detailed enough to ensure that those costs that were likely to be incurred under normal programme conditions were identified and quantified and the sensitivity analysis modeled the effect of the research team on IPTi costs.

The cost findings of the key components were consistent with costing studies of other interventions, although different methodologies were used and different types of costs presented. For example, as shown by Mulligan and colleagues (2006)[10], we found that policy related costs are mainly incurred at national level and that training accounts for a significant proportion of the total cost at district level. Other studies have reported that national level costs for administration and material purchase can constitute a considerable proportion of scale-up costs in the short term [9] and their inclusion increases overall intervention costs. It is therefore important to consider costs of administration when estimating resource requirements for health intervention scale-up.

The unit cost per dose delivered is very low compared with other interventions such as Insecticides Treated Nets (ITNs) or Indoor Residual Spraying(IRS)[25,26]. In this study the cost per IPTi-year of protection (3 doses) was US 69 Cents while the cost per net-year of protection ranged from US$1.43 to US$6.05 and the cost per IRS-year of protection ranges from US$3.27 to 3.90[27]. However, caution is needed in making a direct comparison as the efficacy of various interventions differs and the costing studies used different methodologies. The present study, however, was inclusive in the costs identified and measured, and hence conclusions on the low unit cost of IPTi relative to unit costs of other malaria control interventions are likely to be robust.

Delivery of the intervention at facilities involved no financial resources because excess system capacity was utilized. In effect the intervention boosted efficiency of resource use, while it did not appear to add undue strain to the health system, according to a time and motion study conducted on vaccinating staff in the IPTi study area[28]. Interestingly, IPTi required very few marginal resources even when administration costs were included. To establish and implement the IPTi strategy would require 0.4% of the total health sector budget for 2004/5. Furthermore, only 1% of a typical district budget would be required to develop and run the IPTi intervention in its first year. This might be affordable given the increased global resources to fight HIV, TB and malaria .

The cost of IPTi implementation is likely to vary between countries but the estimates in this paper provide an order of magnitude of the resources required to initiate and maintain implementation of IPTi in most sub-Saharan African settings, especially those with high EPI coverage of 80 per cent or more . Costs vary between settings and change over time, such as the level of staff remuneration, travel expenses and drug costs. What has been presented provides a starting point that could be considered in the light of available system resources and feed into rational decision making.

Scaling-up health interventions is essential if the millennium development goal for child mortality (target 4) is to be met. Strengthening of health system components – human resources, planning capacity, financing and service delivery – is one response to alleviate challenges to move forward to scale-up interventions. Cost data have been presented in a synthesized manner to facilitate utilization by users[29]. The distinction between financial and economic perspectives has implications for health system resource allocation to achieve greater efficiency. The financial cost has a direct influence on budgetary actions and the economic perspective is useful when considering efficient allocation and re-allocation of resources, especially those already available and paid for by the health system. These different cost categories were presented to support interpretation of total costs and eventual use of the information by decision makers. The measurement of full costs and the distinction between cost categories is important because, although some costs might not require budget expenditure in one setting, they might do in another setting due to variation between health systems.

## Conclusion

Actual country-specific data is useful to indicate orders of magnitude of resource requirements and should facilitate rational decision making. The incremental financial and opportunity costs needed to initiate and implement IPTi at national scale show that the intervention is generally affordable within the budget constraints of Ministries of Health of sub-Saharan African countries with high EPI coverage.

## Abbreviations

CHMT: Council Health Management Teams; MoHSW: Ministry of Health and Social Welfare; DPT: Diphtheria Pertussis Tetanus; EPI: Expanded Programme on Immunization; IPTi: Intermittent Preventive Treatment in infants; MCH: Maternal and Child Health.

## Competing interests

The authors declare that they have no competing interests.

## Authors' contributions

FM conceived the idea and participated in the design of the study, conducted the analysis and writing the manuscript. GH participated in the design of the study, provided technical support and contributed to the manuscript preparation. JS participated in conceiving the idea, study design and writing and interpretation. PA, HM, MT provided technical support. DS participated in the design of the study, coordinated the study, data analysis and interpretation. All authors read, commented on and approved the manuscript.

## Pre-publication history

The pre-publication history for this paper can be accessed here:



## Supplementary Material

Additional File 1 Additional table 1: Estimated resources cost for IPTi strategy development and first year implementation **per district **as part of a national program. Figures are United States dollars, year 2005 (Tsh1205 = US$1) Click here for file
