# Supplementary material for: From strategy development to routine implementation: the cost of Intermittent Preventive Treatment in Infants for malaria control
Source: BMC Health Serv Res. 2008 Jul 31;8:165. doi: 10.1186/1472-6963-8-165 (PMC2527562; doi:10.1186/1472-6963-8-165)
Supplement: Additional File 1 — Additional table 1: Estimated resources cost for IPTi strategy development and first year implementation per district as part of a national program. Figures are United States dollars, year 2005 (Tsh1205 = US$1) [file 1472-6963-8-165-S1.doc]

Additional table 1: Estimated resources cost for IPTi strategy development and first year implementation **per district** as part of a national program. Figures are United States dollars, year 2005 (Tsh1205=US$1)

| **Activities** | **Level** | **Human Resource** | |  | **Transport** | | | **Material and building** | | | **Total** | | |
| --- | --- | --- | --- | --- | --- | --- | --- | --- | --- | --- | --- | --- | --- |
|  |  | ***Opport.^a^ cost*** | ***Financ.^b^ cost*** | ***Sub-total*** | ***Opport.^a^ cost*** | ***Financ.^b^ cost*** | ***Sub-total*** | ***Opport.^a^ cost*** | ***Financ.^b^ cost*** | ***Sub-total*** | ***Opport.^a^*** | ***Financ.^b^*** | ***Grand*** |
| **Development costs (start-up)** | | | | | | | | | | | | | |
| Policy change | National^c^ | 39 | 6 | 45 | 2 | 3 | 5 | 0 | 6 | 6 | 41 | 15 | 56 |
| Sensitization | National^c^ | 3 | 0 | 3 | 0 | 0 | 0 | 0 | 0 | 0 | 3 | 0 | 3 |
|  | District^d^ | 899 | 650 | 1,549 | 635 | 349 | 984 | 12 | 54 | 65 | 1,546 | 1,052 | 2,598 |
| BCC | National^c^ | 104 | 55 | 158 | 3 | 4 | 7 | 0 | 10 | 10 | 106 | 69 | 175 |
|  |  |  |  |  |  |  |  |  |  |  |  |  |  |
| Training | District^d^ | 1,442 | 2,843 | 4,285 | 1,729 | 1,018 | 2,746 | 2 | 359 | 360 | 3,172 | 4,220 | 7,392 |
|  |  |  | |  |  |  |  |  |  |  |  |  |  |
| **Implementation costs (annual recurrent)** | | | | | | | | | | | | | |
| Drug purchase & distribution | National^c^ | 72 | 0 | 72 | 0 | 139 | 139 | 0 | 3,328 | 3,328 | 72 | 3,466 | 3,538 |
| Administration of intervention | District^d^ | 345 | 0 | 345 | 0 | 0 | 0 | 0 | 0 | 0 | 345 | 0 | 345 |
| Management & monitoring | National^c^ | 43 | 193 | 236 | 0 | 68 | 68 | 0 | 5 | 5 | 43 | 266 | 309 |
|  | District^d^ | 0 | 0 | 0 | 0 | 0 | 0 | 0 | 170 | 170 | 0 | 170 | 170 |
|  |  |  |  |  |  |  |  |  |  |  |  |  |  |
| **Total** | ***National^c^*** | ***260*** | ***253*** | ***514*** | ***5*** | ***214*** | ***219*** | ***0*** | ***3,349*** | ***3,349*** | ***265*** | ***3,816*** | ***4,081*** |
| **Total** | ***District^d^*** | ***2,686*** | ***3,493*** | ***6,179*** | ***2,364*** | ***1,367*** | ***3,730*** | ***14*** | ***583*** | ***595*** | ***5,063*** | ***5,442*** | ***10,505*** |
| **Grand Total** | **All** | **2,946** | **3,746** | **6,693** | **2,369** | **1,581** | **3,949** | **14** | **3,932** | **3,944** | **5,328** | **9,258** | **14,586** |

^a^Opport. – Opportunity ^b^Financ. – Financial

National**^c^** – Apportioned cost **per district** for national level activities District**^d^** – Cost per district for activities at district level or below
